# Zinc enhances temozolomide cytotoxicity in glioblastoma multiforme model systems

**DOI:** 10.18632/oncotarget.11382

**Published:** 2016-08-19

**Authors:** Amos Toren, Tatyana Pismenyuk, Michal Yalon, Shani Freedman, Amos J. Simon, Tamar Fisher, Itai Moshe, Juergen K.V. Reichardt, Shlomi Constantini, Yael Mardor, David Last, David Guez, Dianne Daniels, Moria Assoulin, Ruty Mehrian-Shai

**Affiliations:** ^1^ Pediatric Hemato-Oncology, Edmond and Lilly Safra Children's Hospital and Cancer Research Center, Sheba Medical Center, Tel Hashomer Affiliated to The Sackler School of Medicine, Tel-Aviv University, Tel Aviv, Israel; ^2^ YachayTech University, Urcuquí, Ecuador; ^3^ Department of Pediatric Neurosurgery, Dana Children's Hospital, Tel-Aviv-Sourasky Medical Center, Israel; ^4^ The Advanced Technology Center, Sheba Medical Center, Tel Hashomer Affiliated to The Sackler School of Medicine, Tel-Aviv University, Tel Aviv, Israel

**Keywords:** glioblastoma multiforme, temozolomide, chemosensitivity, proliferation, zinc

## Abstract

Temozolomide (TMZ) is an alkylating agent that has become the mainstay treatment of the most malignant brain cancer, glioblastoma multiforme (GBM). Unfortunately only a limited number of patients positively respond to it. It has been shown that zinc metal reestablishes chemosensitivity but this effect has not been tested with TMZ. Using both *in vitro* and *in vivo* experimental approaches, we investigated whether addition of zinc to TMZ enhances its cytotoxicity against GBM. *In vitro* cell viability analysis showed that the cytotoxic activity of TMZ was substantially increased with addition of zinc and this response was accompanied by an elevation of *p21, PUMA, BAX* and Caspase-3 expression and a decrease in growth fraction as manifested by low ki67 and lower colony formation. Analysis of GBM as intracranial xenografts in athymic mice and administration of concurrent TMZ and zinc yielded results consistent with those of the *in vitro* analyses. The co-treatment resulted in significant reduction in tumor volume in TMZ/zinc treated mice relative to treatment with TMZ alone. Our results suggest that zinc may serve as a potentiator of TMZ therapy in GBM patients.

## INTRODUCTION

Glioblastoma multiforme (GBM) is the most common and aggressive primary malignant brain tumor [1]. Current treatment of GBM consists of a multi-modality approach including surgery, radiotherapy, and chemotherapy [2]. Temozolomide (TMZ) is a DNA damaging chemotherapeutic alkylating agent which induces apoptosis, senescence and activation of stress mechanisms in brain cancer cell lines [3], [4]. TMZ has become an integral part of malignant GBM management because of its proven efficacy, ease of administration, and favorable toxicity profile [5]. Unfortunately, the efficacy of TMZ has proven to be somewhat limited. In patients with newly diagnosed GBM, the median increase in survival is only 2.5 months compared with radiotherapy alone [6].

Chemotherapy, including TMZ, damages DNA and activates the *TP53* tumor suppressor gene to directly induce the expression of target genes involved in cell-cycle arrest, DNA repair, senescence and, importantly, apoptosis [7]. Inactivation of p53 can result in resistance to apoptosis and failure to respond to DNA-damaging agents like TMZ [8]. *TP53* is mutated in a wide range of human cancer cells [9, 10]. In the presence of DNA damage, p53 is activated as a transcription factor to directly induce the expression of target genes involved in cell-cycle arrest, DNA repair, senescence and apoptosis [11]. Abrogation of wild-type p53 function results in resistance to apoptosis and strongly attenuates TMZ cytotoxicity. Conversely, the p53 wild-type conformation sensitizes glioma cells to the cytotoxic effects of TMZ [12]. It is known that abrogation of p53 wild-type function results in failure to respond to DNA-damaging agents [13]. Therefore, *TP53* mutations may attenuate TMZ cytotoxicity. Conversely, the CP-31398 small molecule which stabilizes and restores p53 function in p53 mutant LN-18 cells, sensitizing glioma cells to TMZ cytotoxicity [14].

It has been shown that zinc is essential for proper p53 function. p53 binds to DNA through a structurally complex domain that is stabilized by zinc cation (Zn^2+^) [15]. In a mammary tumor mouse model it has been shown that zinc restores wild-type p53 conformation and enhances the chemotherapeutic efficacy of adriamycin [16, 17]. Moreover, the addition of ZnCl_2_ reestablished chemosensitivity of adriamicyn and cisplatin in the breast cancer SKBR3 and GBM U373MG cell lines by restoring the p53 active conformation and consequent induction of pro-apoptotic transcription activity [18]. Another study has indicated that the supplementation of zinc allowed to reduce the amount of adryamicin in *in vivo* and *in vitro* colon cancer cells and still have efficient cytotoxic effect [19].

In this study, we investigated the effect of zinc addition on GBM tumor cell toxicity in conjunction with TMZ since it is the standard clinical treatment for GBM.

## RESULTS

### The combination of TMZ and ZnCl_2_ is more cytotoxic than TMZ only to GBM cell lines

**Figure 1:**
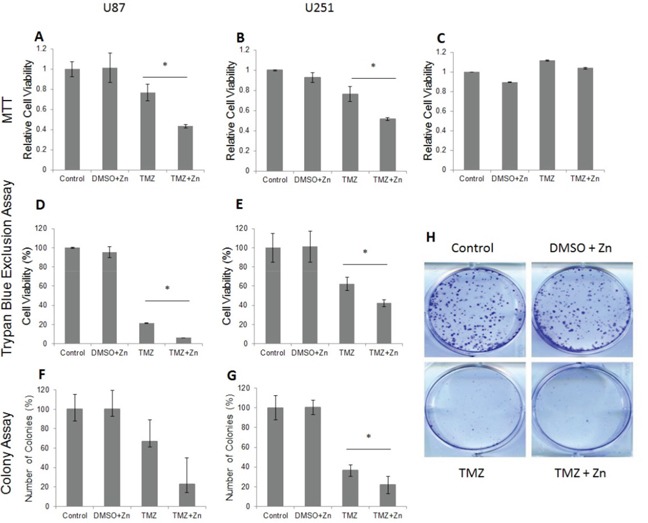
The addition of ZnCl2 to TMZ (combined treatment) reduces the cell viability in U87-MG and U251-MG cell lines as demonstrated in MTT assay, Trypan blue exclusion and colony formation assays **A.** U87-MG MTT assay results **B.** U251-MG MTT assay results. The MTT values were normalized to control. The Y-axis represents the percentage of cell viability whereas the X-axis represents the treatment. Data is reported as means ± SD of 6 repeats and statistical significance was determined by one-way ANOVA. * - P<0.05 compared to TMZ. **C.** The addition of ZnCl_2_ has no effect on cell viability in a normal human astrocyte (NHA) cell line. The MTT values were normalized to control. Experiments were repeated in triplicates. The Y-axis represents the percentage of cell viability whereas the X-axis represents the treatment. Data is reported as means ± SD of 6 repeats and statistical significance was determined by one-way ANOVA. No significant difference between groups was detected. P>0.05 for all groups. **D.** Trypan blue exclusion assay results for U87-MG, **E.** Trypan blue exclusion assay results for U251-MG. The number of cells was evaluated and standardized to control that was set to 100%. The Y-axis represents the percentage of cell viability whereas the X-axis represents the treatment. Data is reported as means ± SD of triplicates and statistical significance was determined by one-way ANOVA. * - P<0.05 compared to TMZ ** - P<O.001 compared to DMEM and DMSO +Zn. **F.** U87-MG colony formation results. **G.** U251 colony formation results. Representation of the quantified cell number. The Y-axis represents the percentage of counted number of colonies whereas the X-axis represents the treatment. The colonies were counted by using ImageJ software. Data is reported as means ± SD of triplicates and statistical significance determined by T test for independent samples.*- P<0.05 compared to TMZ. **H.** Scanned image of 6-well plate colony formation assay in U251-MG.

In order to evaluate the effect of the TMZ and ZnCl_2_ (Zn) combined treatment on cell viability, several tests were performed. First, the viability was evaluated by MTT assay for U87-MG and U251-MG cell lines (Figure 1A and 1B). For U87-MG (wild-type p53), ZnCl_2_ addition to TMZ almost doubled the amount of cell death (Figure 1A) compared to treatment with TMZ only. For U251-MG (mutant p53), addition of ZnCl_2_ to TMZ impact was lower (1.5 times more cell death) and was achieved later (109 h) than in the U87-MG cells (48 h) (Figure 1B). ZnCl_2_ and DMSO (the TMZ solvent) had no significant impact on cell viability (Figure 1A and 1B). We also tested the effect of TMZ and zinc on another GBM cell line, T98G. As expected, TMZ+/−ZnCl_2_ had no effect on T98G since it has an active DNA damage repair O6-methylguanine-methyltransferase MGMT [20] in contrast to U87-MG and U251-MG cell lines in which the MGMT promoter is methylated [21] (data not shown). In order to exclude the possibility of ZnCl_2_ toxicity on normal glial cells, we also performed MTT assays on a normal human astrocyte (NHA) cell line. Addition of ZnCl_2_, DMSO, TMZ or TMZ and ZnCl_2_ showed no effect on cell viability of NHA compared to NHA cells without treatment (Figure 1C). Trypan Blue exclusion assay also revealed higher cytotoxicity when ZnCl_2_ was added to TMZ treatment in both U87 and U251-MG cells (Figure 1D and 1E). Cellular viability in U87-MG was reduced from 22% with TMZ only to 6% with TMZ and ZnCl_2_ (Figure 1D) and from 62% to 42% in U251-MG (p<0.05) (Figure 1E). Colony formation assay was used to determine the effect of the combined treatment on proliferation and survival of treated cells (Figure 1F, 1G and 1H). For U87-MG, the number of colonies was reduced significantly with the addition of ZnCl_2_ vs TMZ only (23 vs 67) (Figure 1F). Although less striking, the addition of ZnCl_2_ to TMZ also reduced significantly the formation of colonies in U251-MG cells (22 vs 36; Figure 1G). As shown above, all three cell viability assays indicated a greater effect of ZnCl_2_ when added to TMZ in the U87-MG cell line compared with the U251-MG cell line. In fact, we have shown previously that even though the *TP53* gene is not mutated in U87-MG cell line, the conformation of the p53 protein is non active because of high levels of metallothionein which reduce the levels of bioavailable zinc [22]. We have also reported that in U87-MG cells, p53 conformation changes to the active form as a result of ZnCl_2_ addition [22]. Thus, zinc addition is more effective on p53 when the inactive conformation is the result of zinc deficiency as the case is with U87-MG.

### The combination TMZ+ZnCl_2_ treatment cytotoxicity is accompanied by increased *PUMA* and *BAX* pro-apoptotic gene expression, and caspase 3 activity

**Figure 2:**
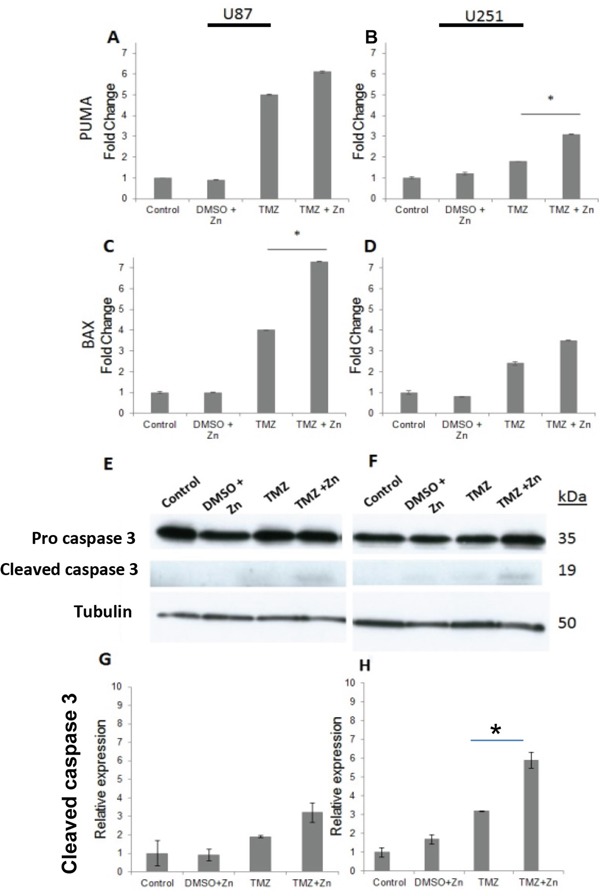
The addition of ZnCl2 to TMZ elevates the levels of *PUMA, BAX* and Caspase-3 **A.** Relative expression of *PUMA* in U87-MG, **B.** Relative expression of *PUMA* in U251-MG. The X axis represents the treatment, whereas the Y axis represents the relative expression of *PUMA*. Data is reported as means ± SD of triplicate and statistical significance was determined by one-way ANOVA. *- P<0.05 compared to TMZ and control,.**C.** Relative expression of *BAX* in U87-MG, **D.** Relative expression of *BAX* in U251-MG. The X axis represents the treatment, whereas the Y axis represents the relative expression of *BAX*. Data is reported as means ± SD of triplicate and statistical significance was determined by one-way ANOVA. *- P<0.05 compared to TMZ and control. **E.** U87-MG expression of the precursor caspase 3, active caspase 3 and tubulin. **F.** U251-MG western blot expression of the precursor caspase 3, active caspase 3 and tubulin. The blot represents caspase-3 levels, 38h for U251-MG and 12h for U87-MG, following treatment. The name of the antibodies is indicated at the left, whereas the molecular weight is shown on the right. 35 kDa indicates the molecular weight of the precursor caspase-3, whereas 19 kDA indicates the active form of caspase-3. Tubulin serves as a house keeping gene control and is indicated by 50 kDa. **G.** U87-MG, **H.** U-251-MG Quantification of active caspase-3 level compared to no treatment control. The X-axis represents the different treatments, whereas Y-axis represents relative band density. The densitometry bar results of the Western blots represent means ± SD of triplicates.

In order to test whether the addition of ZnCl_2_ results indeed in the reactivation of p53, expression analysis was performed for the p53 target genes *PUMA* and *BAX*. Elevation of expression in these genes would also suggest apoptosis as the mechanism of cell viability reduction of both, U87-MG and U251-MG cell lines. In both cell lines, the combined treatment induced the expression of the pro-apoptotic genes to higher levels when compared to TMZ treatment alone (Figure 2A, 2B, 2C and 2D). Significant increase in *PUMA* expression level was detected in U251-MG cells, P<0.05 (Figure 2B) whereas in U87-MG a significant increase was detected in *BAX* gene expression, P<0.05 (Figure 2C). To confirm that the cellular death mechanism is due to apoptosis, we quantified the active form of caspase-3, a classical marker for apoptosis, using western-blot analysis (Figure 2E and 2F). Increased levels of cleaved caspase-3 were seen in the combined TMZ+ZnCl_2_ treated cell lines confirming that cell death is indeed due to apoptosis (Figures 2G for U87 and 2H for U251-MG).

### Cells that escape apoptosis express less Ki67 proliferation marker and are in a senescent state after the combined treatment

**Figure 3:**
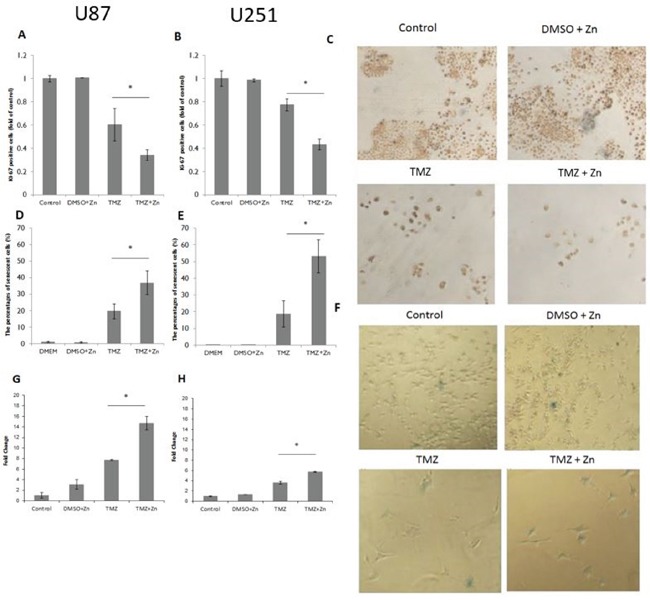
Ki-67proliferation marker, Beta-galactosidase activity and *p21* expression after combination treatment **A.** Immunostaining for Ki-67 in U87-MG cells. **B.** Immunostaining for Ki-67 in U251-MG cells. The Y-axis represents the percentage of Ki-67 positive cells whereas X-axis represents the treatment. Data is reported as means ± SD of triplicates and statistical significance was determined by one-way ANOVA.. * - P<0.05 compared to TMZ. **C.** Representative photomicrographs showing marked Ki-67 staining in U251-MG cells. Cells stained over 50% were considered as positive cells. **D.** Beta-galactosidase activity detection in U87-MG cells to detect senescence. **E.** Beta-galactosidase activity detection in U251-MG cells. The Y-axis represents the percentage of beta-gal positive cells whereas X-axis represents the treatment. Data is reported as means ± SD of triplicates and statistical significance was determined by one-way ANOVA, * P<0.05, compared to TMZ. **F.** Representative micrographs of U87-MG beta-galactosidase staining. Senescent cells appear as blue-colored cells. **G.** Relative expression of *p21* in U87-MG, **H.** Relative expression of *p21* in U251-MG. X axis represents the treatment, whereas the Y axis represents the relative expression of *p21*. Data is reported as means ± SD of triplicates and statistical significance was determined by one-way ANOVA, *P<0.05, compared to TMZ.

In order to assess the effect of TMZ and ZnCl_2_ combination on cell proliferation, Ki-67 levels were measured. Ki-67 is expressed from early entrance into G1-phase throughout the cell cycle and its immunohistochemical staining correlates with the growth fraction of cells. Following the combined treatment with TMZ and ZnCl_2_, in both U87-MG (Figure 3A) and U251-MG (Figure 3B) cell lines, the number of Ki-67 positive cells decreased significantly, compared to TMZ treatment alone. In U87-MG cells, the addition of ZnCl_2_ reduced the number of Ki-67 positive cells by 66% compared to 40% in TMZ only treated cells (Figure 3A). In U251-MG cells, the addition of ZnCl_2_ reduced the number of Ki-67 positive cells by 57% compared to 33% in TMZ only treated cells (Figure 3B). Representative photomicrographs showing the intensely brown stained cells which are considered as Ki-67 positive are shown in Figure 3C.20X magnification of the photomicrographs showing marked Ki-67 staining in U87-MG cells is Supplementary Figure S1. Thus, after combined treatment with zinc and TMZ, there is a significant reduction in the number of cells due to apoptosis and in addition, the remaining cells proliferate less.

In order to assess the effect of TMZ and ZnCl_2_ combination treatment on cellular senescence, β-galactosidase activity was measured. Following the combined treatment, in both U87-MG and U251-MG cell lines, most of the remaining cells that did not undergo apoptosis are in a state of senescence (blue colored cells) (Figure 3D, 3E and 3F). After activation of p53 by the combination treatment, TMZ can activate the p53-*p21* pathway and induce cell cycle arrest [23]. In order to link the apoptosis effect of the combined treatment we checked by RQ PCR the expression level of *p21*. Indeed, *p21* level rose significantly after the combined treatment compared to TMZ treatment alone. This induction was more significant in U87-MG cell line (Figure 3G and 3H).

### The combination treatment changes U87-MG cell morphology (reduction in volume and diameter) and prevents aggregation (sphere formation) of U87-MG cell line after stress condition

Since U87-MG cells respond the most to ZnCl_2_, cellular morphology analysis was performed on this line (Supplementary Figure S2). The cellular volume and diameter increased with TMZ treatment (Supplementary Table S2) (5.6pl volume, 22μm diameter) and decreased with the TMZ+ZnCl_2_ combination treatment (to a 1.2 pl volume and a 13 μm diameter), compared to cellular morphology without any treatment (1.8pl, 15 μm). Cell Volume Shrinkage is known as a major hallmark of early morphological changes during apoptosis [24] and it is seen after our combined treatment, whereas the process known to underlay *in vitro* senescence, progressive increase in the mean cell volume [25], is seen mostly after treatment with TMZ.

In addition, to simulate the response of these cells in perivascular and hypoxic regions which is known to generate angiogenesis promoting cells and increase survival of cancer cells, we monitored the response of U87-MG cells to starvation stress conditions (culturing the cells without adding fresh medium) without and after treatment. After one week during which the medium was not replaced, the cells without any treatment, and cells treated with DMSO and ZnCl_2_ formed aggregates tending to sphere formation (Supplementary Figure S3). Sphere forming cells are referred to as glioblastoma stem-like cells (GSLCs) demonstrating improved survival subsequent to irradiation and chemotherapy and contribute to the recurrence of GBM [26]. These aggregates were inhibited by both the TMZ and the combination treatment but cells in the TMZ arm are enlarged, as in senescence, and the cells with combination treatment are smaller as cells appear before apoptosis as can be seen in the Supplementary Figure S3. Here again, addition of zinc to TMZ treatment reduced the potential of U87-MG cells to survive under stress conditions and also reduced the tumor dormancy.

### Attenuation of tumor development in glioblastoma xenograft mice treated by TMZ and ZnCl_2_

Since glioblastoma therapy is the primary application of our study, we were interested in testing the antitumor efficacy of the combination treatment in an orthotopic xenograft model of GBM. We chose the U87-MG human GBM cell line to study the therapeutic efficacy of ZnCl_2_ because this line was the most responsive to ZnCl_2_ and it also forms highly aggressive orthotopic tumors in nude mice.

**Figure 4:**
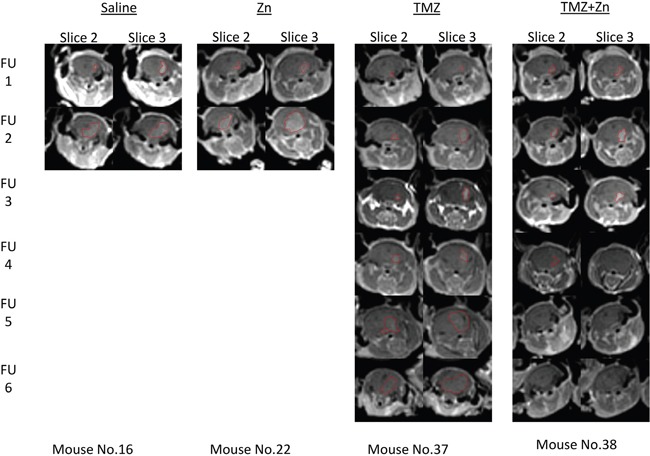
Tumor detection by contrast enhanced T1-weighted MRI of xenograft mice Tumor shrinkage is evident only as a result of ZnCl2 and TMZ combination treatment. The figure represents consecutive MRI scans follow ups (FU) of the same mouse with the specified treatment. Slices 2 and 3 are presented since they had the highest volume at the first MRI and also used for the follow ups. The region of interest (tumor) is indicated with red line.

We explored the ability of orally administered TMZ and zinc to inhibit tumor growth in nude mice carrying intracranial implants of U87-MG cells compared to treatment with TMZ only, ZnCl_2_ only or PBS control. A total of 48 nude mice were stereotactically inoculated with 300,000 U87-MG cells each. After the first MRI, the mice were distributed homogenously into treatment groups according to tumor size. During treatment, five additional MRI scans were conducted in order to evaluate the effect of the treatment on tumor progression. Figure 4 illustrates representative MRI follow ups of one mouse in each treatment and the tumor region circled by red mark. For these, the tumor shrunk only in the mouse with the combined TMZ+ZnCl_2_ treatment.

**Figure 5:**
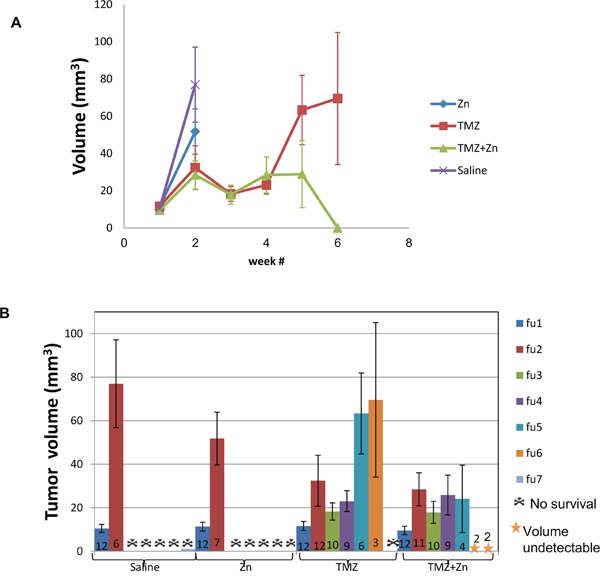
The combination treatment of ZnCl2 and TMZ attenuates and may shrink tumor size in U87-MG xenografts **A.** Total tumor volume in each treatment group. Time points in weeks represent the time points where MRI was imaged. **B.** Mean tumor volume (cubic millimeter) and standard deviation are presented for each group at each time point. Each colored column represents MRI follow-up at a specific time point. The numbers at the bottom of each column represent the number of viable mice at this point. Different colors represent MRI follow-up.

ZnCl_2_ only or PBS treated animals developed substantial tumors from which they died or were terminated by humane sacrifice according to the institutional committee guidelines between 14 and 21 days after tumor cell implantation with no clear difference between these two groups. For TMZ treated mice, 75% of animals were alive after 28 days. 92% of animals treated with ZnCl_2_ in combination with TMZ were alive after 28 days, suggesting that ZnCl_2_ in combination with TMZ may be effective and tumor specific in this aggressive GBM model. In the combination treatment, two of the twelve mice showed no detectable tumor beginning with the third (ie at 21d) MRI and all following MRIs. These mice continued living beyond two months after the completion of the experiment. To investigate this effect further, we quantified the tumor volume. First, we compared the total tumor volume across treatment groups (Figure 5A) and (Supplementary Table S3). A significant reduction of tumor volume was observed at the time of the fifth MRI follow up, or 35d, in the combined TMZ and ZnCl_2_ treatment group compared with TMZ treated mice. The tumor volume in the combined treatment group remained steady and even completely disappeared in two mice whilst the tumors kept growing in the TMZ group. At the fifth time point, the tumor volume was significantly lower in the combined treatment group, compared to TMZ treated group (Fisher's exact test p=0.038). Interestingly, at 6 weeks, the surviving mice undergoing the combined treatment were tumor-free while the tumors in the TMZ-treated mice were still growing. Unfortunately, only 2 mice survived in the combination arm, limiting our statistical analyses at this time point. A high mortality rate (5 mice out of 9) was observed in the TMZ+Zncl2 between the fourth and fifth MRI. Out of the 5 mice only 2 deaths could be associated with high tumor volume. The three other deaths were most probably due to the complications of gavage treatment since their tumor volume was low. Overall Six mice from the TMZ and ZnCl2 treated group and two mice from the TMZ group died regardless of the tumor size, during anesthesia or oral gavage procedure. Complications associated with gavage are known to lead to an increase in morbidity and mortality [27]. Next, we compared mean tumor volume across all treatments. Strikingly, the combination treatment resulted in attenuated tumor growth and even shrinkage in some cases whereas TMZ treatment alone resulted in tumor growth (Figure 5B). Finally, comparisons of the blood count and clinical biochemistry blood tests (creatinine, calcium, phosphorus, urea, glucose, bilirubin, total protein, albumin, globulin, cholesterol, alkaline phosphatase, SGOT, SGPT, sodium, potassium, chloride) were made between control groups and each treated group. No significant differences were found in any of the compared measurements. No gross morphological or structural changes, including edema, necrosis, inflammation, or pigmentation, were observed within all organs in the histopathology study suggesting the treatments were well tolerated in all groups (data not shown).

## DISCUSSION

Growth inhibition and induction of cell death are among the major objectives of anti-cancer therapies. TMZ is used as first-line treatment for GBM [28]. Despite optimal TMZ dosing, GBM prognosis remains poor (median overall survival of 11 months [29] [30]. Thus, despite TMZ's great potential, it has contributed only marginally to the prolongation of life of patients affected by this highly malignant brain tumor. So far all the new treatments aimed at targeting specific pathway molecules have not proven therapeutically effective; thus, TMZ remains the only practical therapy regimen. One of the mechanisms of TMZ action is through the p53 pathway that results in apoptosis. The *TP53* gene is mutated in the tumors of majority of GBM patients making U251-MG cells a good cell line model for these patients. We have shown that in GBM patients without *TP53* mutation, p53 may be inactive due to the high expression of metallothionein [22]. We have also shown that the U87-MG cell line is a good model for patients with high metallothionein and no mutation in *TP53*.

Our present study shows that the combined treatment consisting of TMZ and ZnCl_2_ reduces the number of viable cells and their proliferation ability and induces apoptosis. Furthermore, the addition of ZnCl_2_ resulted in reactivation of mutant conformation of p53 to wild-type. Our data are consistent with recent reports that non-active p53 can be activated by zinc that enhances cytotoxicity in combination with cisplatin which is another alkylating agent [31]. More notably we have shown that the combination of TMZ and ZnCl_2_ is more effective than TMZ treatment only in inhibiting tumor growth in nude mouse. Summary of the volume of tumor for each treatment group and the number (n) of mice that survived up to that follow up MRI show a significant high death rate and volume of tumor for the no treatment and DMSO+ ZnCl_2_ treatment and most importantly a significant reduction in tumor volume p <0.05 in the TMZ+ ZnCl_2_ group compared to TMZ only group after 5 weeks. In addition, in two of the combination group the tumor totally shrunk. On the other hand all mice at the TMZ group the tumor volume only grew and finally no mice survived in this group.

Overall, we tested three models for GBM: U251-MG cells which may represent GBM patients with mutant p53, U87-MG cells which may represent GBM patients with inactive p53, and U87-MG xenograft animal model that represents the tumor itself. In all these models, addition of zinc to TMZ treatment enhanced TMZ toxicity. Furthermore, our data demonstrate that the administered dose of zinc does not affect the normal astrocytes and has no effect on organs or blood chemistry of the xenograft mice. This may be an addition to other benefits of zinc given the fact that in the mouse Alzheimer's disease (AD) model it has been found that ZnCl_2_ supplementation is beneficial in the treatment of AD and decrease amyloid plaques [32].

Our findings suggest a role for the addition of zinc to current TMZ treatment regimens for GBM patients. We demonstrate here for the first time that adding zinc to TMZ enhances the cytotoxicity of TMZ in both *in vitro* and an animal GBM model. This ability of specifically targeting and activating the destruction of brain cancer cells without affecting normal astrocytes (as shown here by our *in vitro* studies) is crucial for the development of targeted therapies designed to enhance the cytotoxicity of TMZ.

## MATERIALS AND METHODS

### Cell lines and culture conditions

The human glioblastoma cell lines U251-MG (mutant p53; expressing R273H mutation) and U87-MG (wild-type p53) were a generous gift of Dr. Gad Lavie (Sheba Medical Center) while normal human astrocytes (NHA) were received as a gift of Prof. Chaya Brodie (Bar-Ilan University). The cell lines have been tested and authenticated before the experiments were established by the Promega PowerPlex 16 HS and analyzed using the 3130xl Genetic Analyzer (Life Technologies) and GeneMapper IDX software U251-MG and U87-MG were cultured in Dulbecco's modified Eagle's medium (DMEM; Gibco) supplemented with 10% fetal bovine serum (FBS) and 1% penicillin/streptomycin. Normal human astrocytes (NHA) served as controls and were cultured in Astrocyte Basal Medium (ABM; Lonza) supplemented with 0.1% rhEGF, 0.25% insulin, 0.25% ascorbic acid, 0.1% GA-1000, 1% L-glutamine, 3% FBS and 1% penicillin/streptomycin. All the cell lines were cultured at 37°C, in a humidified 5% CO_2_ atmosphere.

### Treatment regimen and cell viability assays

Our experiments consisted of four arms: no treatment, DMSO + ZnCl_2_ control, TMZ (Sigma) treatment alone, and TMZ + ZnCl_2_ (Sigma) combined treatment. Cells were seeded in triplicate 24 hours before treatment, in order to allow for full cell adherence. On day 2, only the cells of DMSO and ZnCl_2_ control and TMZ and ZnCl_2_ combination treatment exposed to 100μM ZnCl_2_ for U251-MG cell line and to 75μM ZnCl_2_ for U87-MG cell line. TMZ was dissolved in DMSO at 25 or 50μM before treatment and added at day 3. Thereafter, the plates were incubated for 48 or 109 hours based on a time curve for maximum effect. Different concentrations (25, 50 and 100 μM) of TMZ and ZnCl_2_ were tested before we chose the lowest amount of TMZ with the most effect on cells as lower TMZ treatment will also be important in reducing the side effects of treatment in future. For ZnCl_2_ we chose the concentration which is not toxic to normal astrocytes and glioma cells by itself and has the most effect on glioma cells in combination with TMZ. Cell viability was determined by cell counting, MTT assay (Sigma) and colony formation assay. For cell counting, 0.25×10^4^ cells were seeded into 6-well plates. 109 hours after the TMZ (50 μM) and ZnCl_2_ combined treatments, the number of cells were evaluated by suspension of cells in Trypan Blue dye (Biological Industries) and then counting with a hemocytometer in a light microscope.

For the MTT assay, 0.8×10^3^ cells were plated in 96-well plates and treated with TMZ (50 μM) and ZnCl_2_. After the treatment, as described in the treatment regimen, each well was supplemented with the MTT reagent (5mg/ml PBS) at a volume of 10% of the medium and incubated for 3 hours at 37°C, in a humidified 5% CO_2_ atmosphere. Thereafter, 100μl of 0.04 M HCl in absolute isopropanol was added and then the plate was incubated for a further 15 minutes. Absorbance values were measured in an ELISA Reader at 560 and 650 nm.

For colony formation assays, the cells were seeded in 6-well plates (0.8 × 10^3^ cells cells per well) and treated according to the regimen with 3 sequential treatments of TMZ (25 μM) and ZnCl_2_ combination. 14 days after plating, the medium was removed, the cells were washed with PBS and 750 μl 0.5% in ethanol crystal violet (Sigma) was added for 10 minutes at RT (room temperature). The plates were then rinsed extensively in tap water, dried and photographed with a light microscope.

### RNA extraction and quantitative real-time polymerase chain reaction (RT-PCR)

Total RNA was extracted from frozen (at −80°C) cell pellets using the RNeasy plus Mini kit (Qiagen). High-Capacity cDNA Reverse Transcription Kit (Applied Biosystems) was used for cDNA synthesis. Relative transcript expression levels were measured by quantitative real-time PCR using a syber green based method. Gene-specific primers (Supplementary Table S1) were designed by using Primer express software (Applied Biosystems). Average fold changes were calculated by differences in threshold cycles (Ct) between pairs of samples. The mRNA levels were normalized to Beta-actin expression.

### Protein extraction and western blot analysis

Protein was extracted from U87-MG and U251-MG cell lines using RIPA lysis buffer with proteinase inhibitor (Santa Cruz, USA). Protein concentration was measured by the method of Bradford using BCA Protein Assay Kit (Pierce). 30 μg of protein mixed with SDS (sodium dodecyl sulfate) loading buffer was loaded per lane, separated by 12.5% SDS-polyacrylamide gel electrophoresis. Caspase 3 protein was detected with Caspase 3 antibodies from rabbit origin (1:1000 dilution, Cell Signaling). α tubulin served as a house keeping control protein (1:200 dilution, Santa Cruz Biotechnology). The secondary antibodies were anti-Rabbit IgG-HRP (1:10000 dilution, Jackson Immuno Research Laboratories), and anti-Mouse IgG-HRP (1:10000 dilution, Jackson Immuno Research Laboratories), respectively. The intensities of the protein bands were quantified with ImageJ software (http://imagej.nih.gov/ij/).

### Senescence and proliferation detection assays

Cellular senescence was detected as previously described by staining for senescence-associated β-galactosidase activity at pH 6.0 [33]. Photographs of 3 representative fields of each triplicate were taken. Afterwards, the cells were counted by imageJ software and the percentage of β-galactosidase positive cells was determined.

For cell proliferation, Ki-67 levels were detected. The treated cells were plated in triplicate on coverslips in 24-well plate and incubated overnight at 4°C with primary Ki-67 antibody (1:500 dilution, ABCAM). Afterwards, the cells were incubated for 1 h with secondary HRP Goat anti rabbit antibodies (1:500, Jackson Immuno Research Laboratories) diluted in PBS. The cells were subjected to DAB substrate kit staining (Vector) and coverslipped with preheated 50^°^C glycergel (Dako) and then viewed under 4-fold magnification in a light microscope. Pictures of 3 representative fields of each triplicate were taken. The cells were counted by the imageJ program and the percentage of Ki-67 positive cells (Ki-67 labeling index) was determined.

### Cellular morphology analysis

Cells were cultured and treated as described above in the treatment regimen section. The cell suspension was diluted so that the cell concentration was 1×10^6^ cells/ml and a 75μl sample was loaded on a Moxi automated cell counter cassette (Orflo Technologies). A curve-fitting approach and standardized Moxi Viability Index (MVI) analysis was used to determine cell counts [34]. The cellular size distribution, mean cell size and cell volume were recorded. The MVI analysis reflects a population index or ratiometric measure of the monodisperse population counts without particle debris.

### Cellular stress by starvation

Cells were cultured and treated as described above in the treatment regimen section. The cells were introduced to the starvation stress by not changing their culture medium for additional seven days after the 106 hours since treatnent. After one week the plate was analyzed using an inverted optical microscope, in which the photographs were taken for cell morphological analysis.

### *In vivo* tumor xenograft model

Five-week-old male nude mice (Harlen laboratories Inc., Israel) were used for *in vivo* assays. Experiments were approved by the Tel-Aviv University Animal Care and Use Committee (Permit Number 6366). All efforts were made to minimize suffering of the animals. Mice were anesthetized and inoculated with 3 × 10^5^ U87-MG cells using a stereotactic head frame. A small craniotomy was drilled 2 mm to the right of midline and 1 mm anterior to the coronal suture, and cells were injected stereotactically into the right frontal lobe 3 mm below the dura mater. The injection needle was removed, the craniotomy was sealed with bone wax, and the scalp was closed with sutures. Animals were observed daily for appearance and behavioral changes and symptom-free animals were euthanized on day 47. One week after injection, magnetic resonance imaging (MRI) scans were performed and the mice were randomized into four groups (12 mice per group) to receive -by gavage- saline, ZnCl_2_, TMZ alone or the combined treatment consisting of TMZ (82.5 mg/kg) and ZnCl_2_. We used oral gavage (dosing) to make sure all mice got the exact volume of treatment administered orally including TMZ treatment which is given orally to GBM patients. The regimen of treatment for the *in vivo* experiment is described in Table 1. During the treatment period, 5 MRI scans (one scan per week) were conducted in order to evaluate the effect of the treatment. The tumor volume (in mm^3^) was calculated from contrast-enhanced T1-weighted MRI after IP injection of gadolinium diethylenetriaminepentacetate (Gd-DTPA) by defining the region of interest over the entire enhancing region in each slice. The tumor volume was calculated by counting the number of pixels in the regions of interest and multiplying the number by the volume of a single pixel. The tumor growth rate was calculated as the ratio between the tumor volume measured in a certain follow-up (FU) scan and the tumor volume measured in the first follow-up scan. In addition, 400-μl retro-orbital sinus blood was collected for chemistry panel analysis by Hitachi modular-800 (Roche) system (Creatinine, Calcium, Phosphorus, Urea, Glucose, Bilirubin, Total Protein, Albumin, Globulin, Cholesterol, Alkaline Phosphatase, SGOT, SGPT, Sodium, Potassium, and Chloride). Heart, kidney and liver organs tissue samples from euthanized mice were preserved in 10% neutral buffered formalin at the time of sacrifice processed and evaluated by a board-certified veterinary pathologist for gross pathology.

**Table 1:** Regimen of treatment for the *in vivo* experiment

Day of treatment	Group 1: Saline	Group 2: Zn	Group 3: TMZ	Group 4: TMZ and Zn
1	Saline (50 ul)	Zn	Saline (50 ul)	Zn
2-6	Saline (200 ul)	Zn	TMZ	TMZ+Zn
7-13	Saline (50 ul)	Zn	Saline (50 ul)	Zn
25-29	X	X	TMZ	TMZ+Zn
30-37	X	X	Saline (50 ul)	Zn

### Statistics

One-Way ANOVA with post hoc Tukey test (for multiple comparison), or t-test for independent samples (for two groups comparison) were used to evaluate the significance of *in vitro* results. For the complete *in vivo* experiment, Fisher's exact test, Mann-Whitney, Wilcoxon and Log Rank tests were used. A *P*-value of <0.05 was considered significant.

## SUPPLEMENTRY FIGURES AND TABLES



## Abbreviations

GBM: Glioblastoma multiforme
TMZ: Temozolomide
ZnCl_2_: cZinc chloride
