# Supplementary material for: Zinc enhances temozolomide cytotoxicity in glioblastoma multiforme model systems
Source: Oncotarget. 2016 Aug 19;7(46):74860–71. doi: 10.18632/oncotarget.11382 (PMC5342707; doi:10.18632/oncotarget.11382)
Supplement: Supplementary file 1 [file oncotarget-07-74860-s001.pdf]

Zinc enhances temozolomide cytotoxicity in glioblastoma multiforme model systems

SUPPLEMENTRY FIGURES AND TABLES

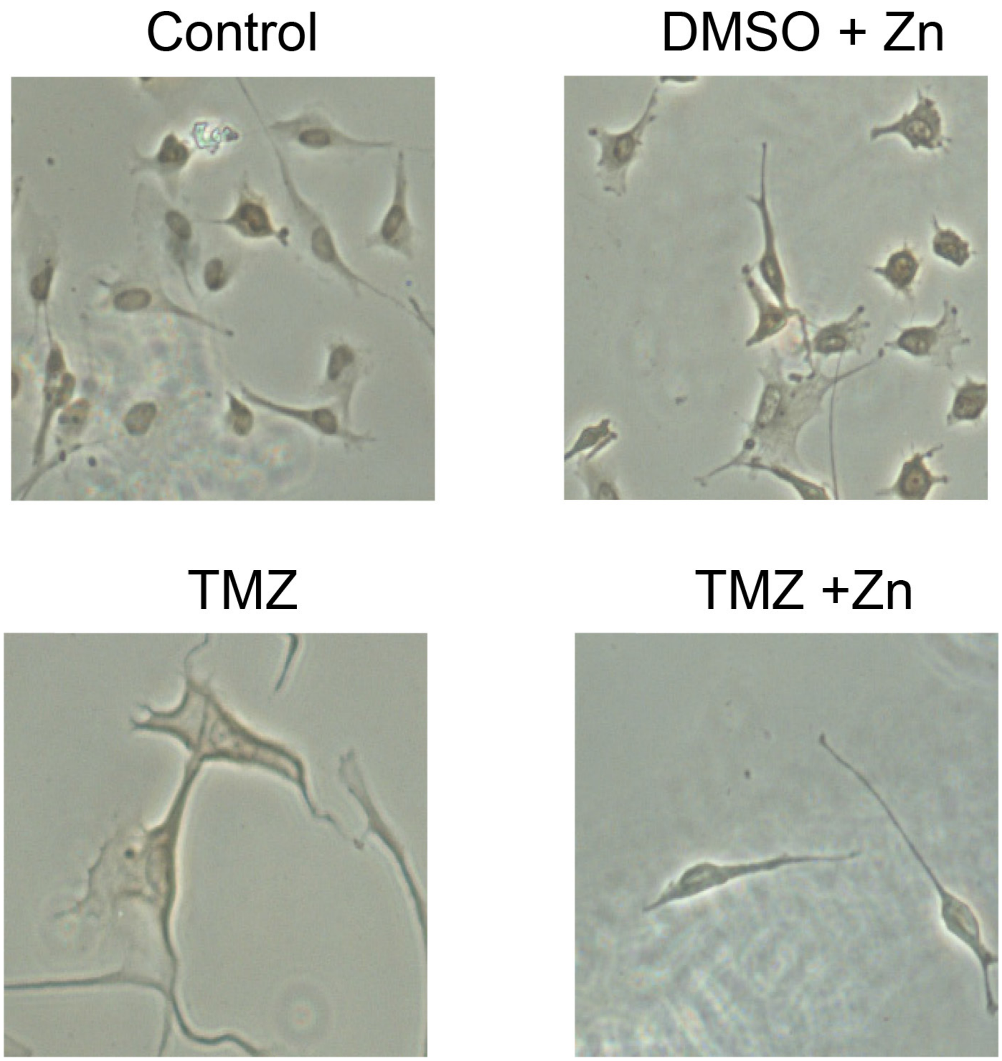

Supplementary Figure S1: 20X magnification of the photomicrographs showing marked Ki-67 staining in U87-MG cells.

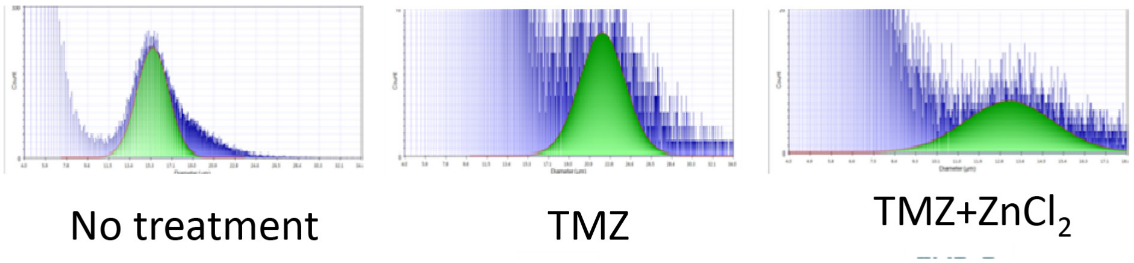

Supplementary Figure S2: U87-MG Cellular morphology histogram before and after TMZ or the combination treatment. The histogram displays a curve fit count on a diameter scale. The cell diameter profiles are drawn in proportion to their contributions to the total viable cell count as result of curve-fitting approach and standardized Moxi Viability Index (MVI) analysis.

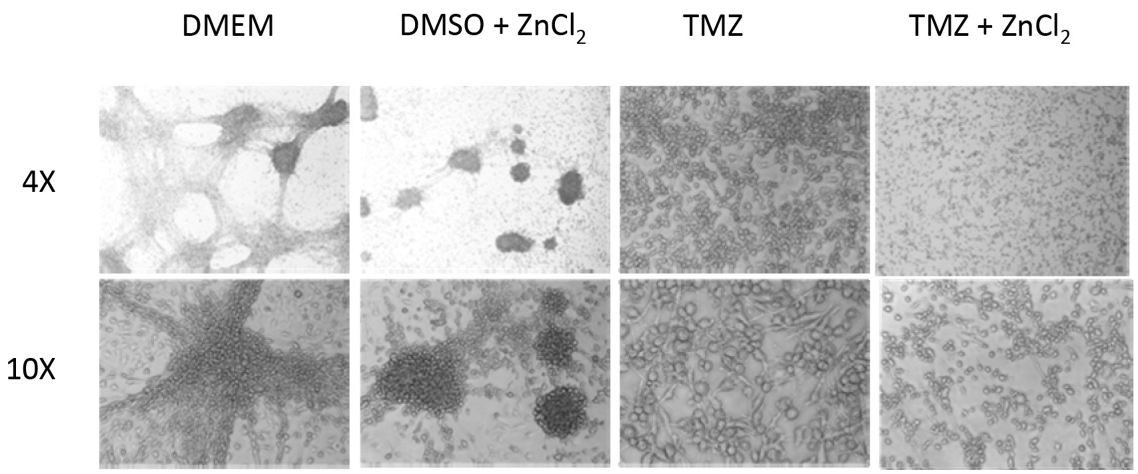

Supplementary Figure S3: Representative photomicrograph of U87-MG cell line after starvation of untreated, treated with DMSO+Zn, TMZ only or combination treatment imaged by 4X and 10X magnification.

Supplementary Table S1: RT-PCR primers sequence

MT3-F	acctcctgcaagaagagctg
MT3-R	cagctgcacttctctgcttct
P21-F	ccttccagctcctgtaacatact
P21-R	aacgggaaccaggacacatg
PUMA-F	gacgacctcaacgcacagta
PUMA-R	gtaagggcaggagtcccat
BAX-F	ccttttctacctttgccagcaaac
BAX-R	gaggccgtcccaaccac
Beta Actin-F	cctggcacccagcacaat
Beta Actin-R	gccgatccacacggagtact

Supplementary Table S2: U87-MG cellular diameter and without and after treatments

No Treatment	TMZ	TMZ+Zn
Diameter: 15 µm	Diameter: 22 µm	Diameter: 13 µm
Volume: 1.8 pL	Volume: 5.6 pL	Volume: 1.2 pL

Supplementary Table S3: Tumor volume in different time points in tumor-bearing mice

	FU1		FU2		FU3		FU4		FU5		FU6		FU7	
**Group**	**n**	**V**	**n**	**V**	**n**	**V**	**n**	**V**	**n**	**V**	**n**	**V**	**n**	**V**
Saline	12	10.48 ± 1.86	6	76.96 ± 20.16	0	NA	0	NA	0	NA	0	NA	0	NA
Zn	12	11.30 ± 2.03	7	51.75 ± 12.14	0	NA	0	NA	0	NA	0	NA	0	NA
TMZ	12	12.08 ± 2.20	12	34.11 ± 12.71	10	19.28 ± 4.27	9	23.01 ± 4.79	6	*63.32 ± 18.57	3	69.53 ± 35.48	0	NA
TMZ+ZnCl2	12	10.39 ± 2.19	11	33.34 ± 8.26	10	19.65 ± 5.24	9	28.86 ± 9.72	4	*28.41 ± 18.0	2	0	2	0

Summary of the volume (V in mm^3, X±S) of tumor for each treatment group and the number (n) of mice that survived and were scanned by MRI in each of the seven weeks follow up (FU). In addition to significant high death rate and volume of tumor for the no treatment and DMSO+zinc treatment, a significant pvalue <0.05 (*) was detected between volume of TMZ and the combination treatment at FU 5. If no mice survived in any follow up the volume is indicated as non applicable (NA).
